# Supplementary material for: Emotional Complications in Midwives Participating in Pregnancy Termination Procedures—Polish Experience
Source: Int J Environ Res Public Health. 2020 Apr 17;17(8):2776. doi: 10.3390/ijerph17082776 (PMC7216072; doi:10.3390/ijerph17082776)
Supplement: Supplementary file 1 [file ijerph-17-02776-s001.pdf]

OLENBURG BURNOUT INVENTORY

Using the scale please indicate, the degree of your agreement or disagreement by selecting the numer that corresponds with your statement.

1________________2________________3________________4

Strongly Agree   Agree   Disagree   Strongly disagree

1.	I always find new and interesting aspects in my work.	1	2	3	4
2.	There are days when I feel tired before I arrive at work.	1	2	3	4
3.	It happens more and more often that I talk about my work in a negative way.	1	2	3	4
4.	After work, I tend to need more time than in the past in order to relax and feel better.	1	2	3	4
5.	I can tolerate the pressure of my work very well.	1	2	3	4
6.	Lately, I tend to think less at work and do my job almost mechanically.	1	2	3	4
7.	I find my work to be a positive challenge.	1	2	3	4
8.	During my work, I often feel emotionally drained.	1	2	3	4
9.	Over time, one can become disconnected from this type of work.	1	2	3	4
10.	Afer working, I have enough energy for my leisure activities.	1	2	3	4
11.	Sometimes I feel sickened by my work tasks.	1	2	3	4
12.	After my work, I usually feel worn out and weary.	1	2	3	4
13.	This is the only type of work that I can imagine myself doing.	1	2	3	4
14.	Usually, I can manage the amount of my work well.	1	2	3	4
15.	I feel more and more engaged in my work.	1	2	3	4
16.	When I work, I usually feel energized.	1	2	3	4

Disengagement items: 1, (3), (6), 7, (9), (11), 13, 15

Exaustions items: (2), (4), 5, (8), 10, 12,14, 16

( ) meanes reversed item when the scores should be such that higher schores indicate more burnout.
